# Exposure of Toluene Diisocyanate Induces DUSP6 and p53 through Activation of TRPA1 Receptor

**DOI:** 10.3390/ijms23010517

**Published:** 2022-01-04

**Authors:** Soee Kim, Min Kim, Jung-Suk Sung

**Affiliations:** Department of Life Science, Biomedi Campus, Dongguk University-Seoul, 32 Dongguk-ro, Ilsandong-gu, Gyeonggi-do, Goyang 10326, Korea; soeesoee@naver.com

**Keywords:** toluene diisocyanate, DUSP6, p53, ERK1/2, TRPA1, cytotoxicity

## Abstract

Toluene diisocyanate (TDI), a major intermediate agent used in the manufacturing industry, causes respiratory symptoms when exposed to the human body. In this study, we aimed to determine the molecular mechanism of TDI toxicity. To investigate the impact of TDI exposure on global gene expression, we performed transcriptomic analysis of human bronchial epithelial cells (BEAS-2B) after TDI treatment. Differentially expressed genes (DEGs) were sorted and used for clustering and network analysis. Among DEGs, dual-specificity phosphatase 6 (DUSP6) was one of the genes significantly changed by TDI exposure. To verify the expression level of DUSP6 and its effect on lung cells, the mRNA and protein levels of DUSP6 were analyzed. Our results showed that DUSP6 was dose-dependently upregulated by TDI treatment. Thereby, the phosphorylation of ERK1/2, one of the direct inhibitory targets of DUSP6, was decreased. TDI exposure also increased the mRNA level of p53 along with its protein and activity which trans-activates DUSP6. Since TRPA1 is known as a signal integrator activated by TDI, we analyzed the relevance of TRPA1 receptor in DUSP6 regulation. Our data revealed that up-regulation of DUSP6 mediated by TDI was blocked by a specific antagonist against TRPA1. TDI exposure attenuated the apoptotic response, which suggests that it promotes the survival of cancerous cells. In conclusion, our results suggest that TDI induces DUSP6 and p53, but attenuates ERK1/2 activity through TRPA1 receptor activation, leading to cytotoxicity.

## 1. Introduction

Toluene diisocyanate (TDI), a highly reactive aromatic compound, is mainly absorbed into the human body through the respiratory tract owing to its volatile properties [1]. TDI absorbed into the body causes inflammation in bronchial cells, thereby causing occupational asthma which leads to various respiratory symptoms such as bronchial irritation, bronchoconstriction, and hypersensitivity pneumonia [2,3,4,5,6,7,8,9,10]. In addition, TDI is classified as a human carcinogen group 2 by the International Agency for Research on Cancer (IARC) [11,12]. It also has been reported that TDI induces gene mutations and chromosomal damage, however, the exact mechanism of carcinogenesis has not been elucidated [13].

TDI is a major intermediate agent in the synthesis of polyurethanes used in the manufacturing industry [14]. Polyurethane foam using TDI as a raw material is utilized in various industrial fields such as automobiles, furniture, and packing materials [5]. Since TDI is known as the main cause of occupational asthma, previous studies on TDI exposure have mainly dealt with its adverse effects and symptoms related to TDI-induced asthma. Previous research has shown that TDI exposure induces not only inflammatory responses but non-inflammatory responses, including oxidative stress generation and airway remodeling [6,8,15,16,17]. On the other hand, the research on the molecular mechanisms after TDI exposure is still insufficient.

The transcriptomics techniques based on next-generation sequencing (NGS) are highly efficient and sensitive techniques that can simultaneously analyze vast amounts of gene expression [18,19]. To reveal the molecular mechanism for underlying complex biological processes, transcriptomic approaches can provide decisive information [20,21,22,23,24]. In this study, we performed an integrative analysis based on transcriptomic profiling to determine what kind of intracellular gene expression pathways are modulated by respiratory exposure of TDI. Among them, the factor that plays a crucial role was selected to elucidate the molecular mechanisms which lead to TDI-induced cytotoxicity. Since TDI mainly impacts the respiratory tract when it is exposed to the human body, it can directly affect bronchial and lung cells. Therefore, we used bronchial epithelial cells (BEAS-2B) to study the toxicity of TDI.

Dual-specificity phosphatase 6 (DUSP6), a member of the MAPKs phosphatase family, specifically interacts with extracellular signal-regulated kinase (ERK) 1/2 and inhibits its activity [25]. Since ERK is a key effector of MAPK involved in the various intracellular responses, the regulation of ERK by DUSP6 could be crucial for controlling intracellular physiological mechanisms [26]. The expression of DUPS6 is regulated by tumor protein p53 (TP53) [27]. Therefore, it is necessary to identify whether the regulation of DUSP6 by TDI is related to the p53 and ERK1/2 pathways.

Generally, the transient receptor potential ankyrin 1 (TRPA1) channel is known to be activated by exogenous stimuli such as cold, heat, and mechanical stimuli, which may produce cellular damage [28]. It has been reported that TDI exposure induces respiratory irritation through activation of TRPA1 [29]. However, the molecular mechanism of TDI exposure, especially p53, DUSP6, and ERK1/2 through TRPA1 activation, has not yet been studied. Therefore, we hypothesized that TDI regulates not only the expression of DUSP6 through TRPA1 activation, but also affects the regulation of p53 and ERK1/2 which are up and downstream genes of DUSP6.

## 2. Results

### 2.1. Selection and Validation of DUSP6 as a Key Molecule Modulated by TDI Treatment

**Figure 1:**
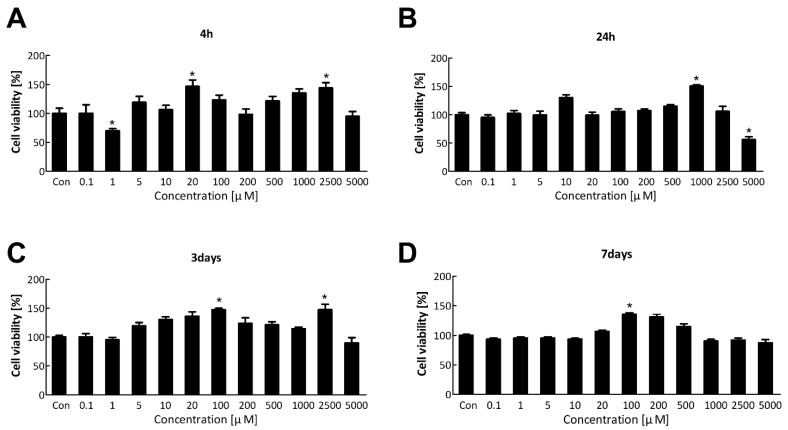
Cell viability after the short-term and long-term exposure to TDI. BEAS-2B cell was treated with various concentrations of TDI for (**A**) 4 h, (**B**) 24 h, (**C**) 3 days, and (**D**) 7 days. Cell viability was measured using the WST assay. Each result shown represents the mean ± standard error of the mean (SEM) taken from at least 5 independent experiments. * *p* < 0.05.

The water-soluble tetrazolium salt (WST) assay was performed to evaluate the cytotoxicity of TDI and select an appropriate concentration of TDI for transcriptomic analysis. Cell viability of BEAS-2B was measured after TDI treatment at various concentrations for 4 h, 24 h, 3 days, and 7 days (Figure 1A–D). TDI did not significantly kill the cells, except after treatment with 5000 μM for 24 h. Therefore, we selected the concentration of TDI which did not affect the cell survival for subsequent experiments; 10 μM for the low concentration and 200 μM for the high concentration.

**Figure 2:**
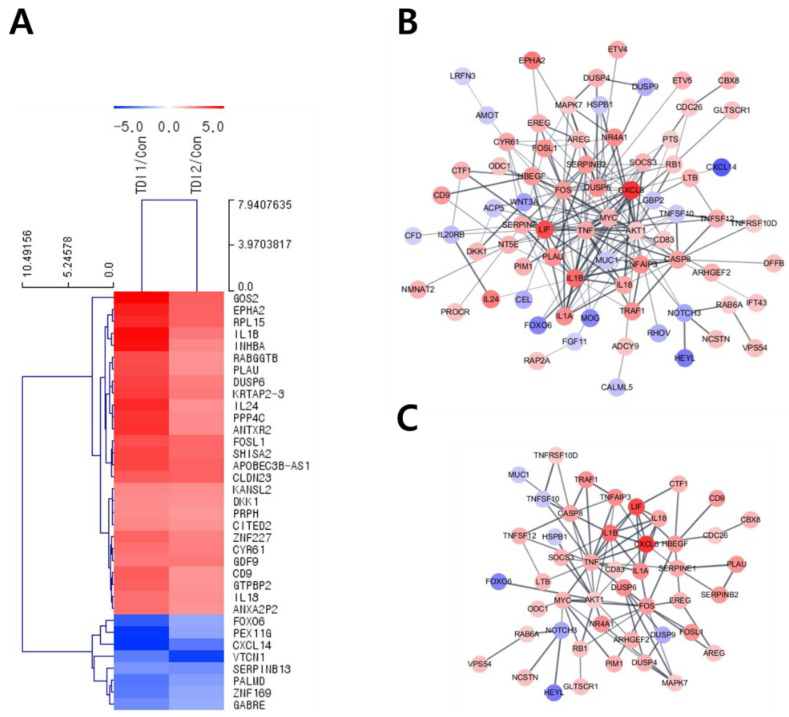
Hierarchical clustering and PPI analysis of differentially expressed genes after TDI exposure. (**A**) Hierarchical clustering analysis was performed using 35 genes with fold changes ≥ 5 and normalized data values ≥ 4 whose expression levels changed significantly with both low and high concentrations of TDI. A MeV software version 4.9 was used for the formation of a Hierarchical Cluster Tree based on normalized fold changes. Colors (red-blue) show relative gene expression changes in TDI1/Con and TDI2/Con. The length of the tree is the distance and the shorter the distance means a similar expression pattern between genes. PPI analysis of differentially expressed genes was investigated by using the STRING database. PPI analysis was conducted using 272 genes with the condition of (**B**) fold changes ≥ 2.5 and normalized data values ≥ 4, and confidence score cut-off of 0.5, and (**C**) values fold changes ≥ 2.5 and normalized data values ≥ 4, and confidence score cut-off of 0.7. Red node and blue node represent significantly increased or decreased genes, respectively. The deeper the interaction between genes, the thicker the line. red: up-regulated, blue: down-regulated genes, TDI1: 10 μM of TDI, TDI2: 200 μM of TDI, Con: control.

**Figure 3:**
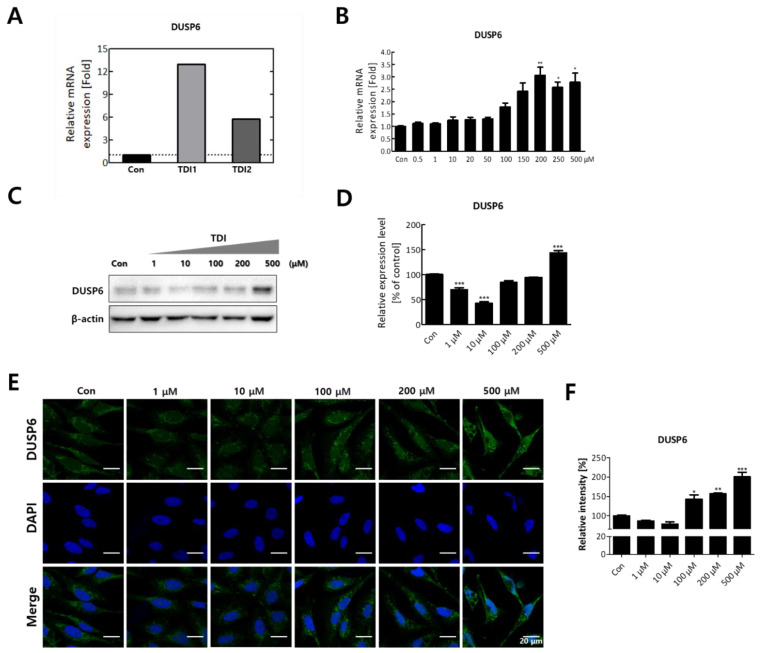
The expression level of DUSP6 after 24h TDI treatment. (**A**) mRNA expression level of DUSP6 in 3’ mRNA quantification sequencing results are represented in a bar graph. (**B**) Gene expression level of DUSP6 was confirmed using quantitative real-time PCR (qRT–PCR). (**C**) The protein expression level of DUSP6 was measured by western blot analysis, and (**D**) the western blot quantification is shown as a bar graph. Each result shown represents the mean ± SEM taken from at least three independent experiments. (**E**) Intracellular DUSP6 and nucleus were stained by CFL 488 and DAPI, respectively. (**F**) The relative fluorescence intensity of DUSP6 was calculated and expressed in a bar graph. * *p* < 0.05, ** *p* < 0.01, *** *p* < 0.001. TDI1: 10 μM of TDI, TDI2: 200 μM of TDI, Con: control.

After treatment with the selected concentration of TDI, mRNA quantification sequencing (Quan-seq) was performed to identify genes whose expression showed significant changes by TDI exposure. DEGs whose expression was commonly modulated by the treatment with low and high concentrations of TDI were classified. To select DEGs with similar comparable expression patterns, hierarchical clustering analysis was performed using sorted genes based on their normalized expression values (Figure 2A). Protein–protein interaction (PPI) was also analyzed using genes significantly modulated by TDI treatment based on STRING and the Cytoscape database (Figure 2B,C). The results with 0.5 of confidence cut-off value showed a complex web of DEGs (Figure 2B). To select the key factors that sensitively responded to TDI exposure, we re-analyzed them by increasing the confidence cut-off value to 0.7 (Figure 2C). The results show that dual-specificity phosphatase 6 (DUSP6) was one of the genes significantly changed by TDI exposure. In the results of the mRNA Quan-seq analysis, the expression levels of DUSP6 increased by 12- and 6-fold after treatment of 10 μM and 200 μM of TDI, respectively (Figure 3A). To validate whether the expression level of DUSP6 increases by TDI in vitro, its mRNA and protein levels were evaluated using TDI-treated-cell extract (Figure 3B–D). Gene expression of DUSP6 was up-regulated in a dose-dependent manner by TDI treatment. In particular, 200 μM of TDI treatment showed the highest expression level, which increased 3-fold (Figure 3B). The protein level of DUSP6 also decreased in the 10 μM treated group and then increased by 1.5-fold in the 500 μM treated group (Figure 3C,D). Intracellular DUSP6 intensity analyzed by Immunocytochemistry (ICC) also decreased in the 10 μM treated group and then increased by 2-fold in the 500 μM treated group (Figure 3E,F).

### 2.2. TDI Regulates DUPS6 by Up-regulation of p53 and Thereby Down-regulation of ERK1/2

**Figure 4:**
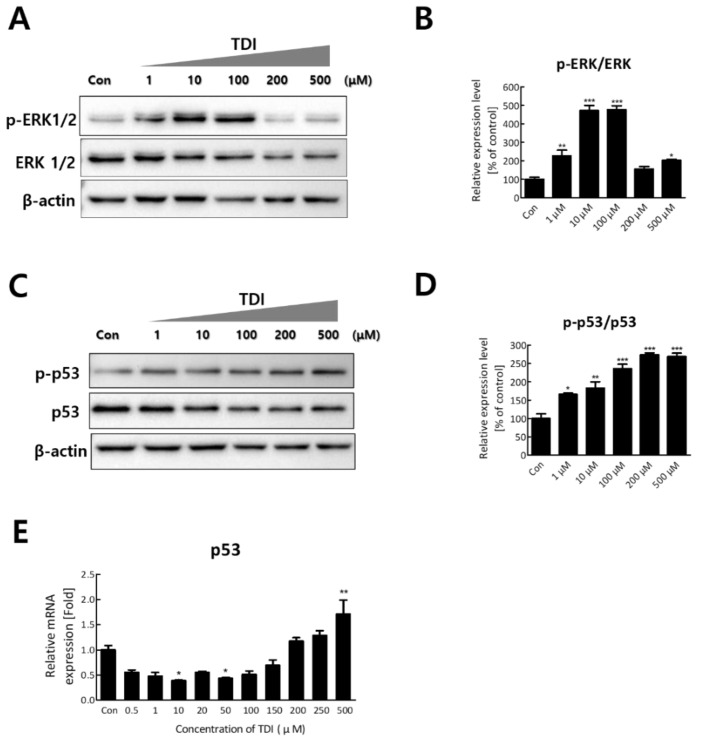
Expression level and activity of p53 and ERK1/2 after TDI treatment for 24h. (**A**) Total proteins were isolated and subjected to immunoblotting with antibodies against p-ERK1/2 and ERK 1/2. (**B**) The activity of ERK1/2 was calculated by quantitation of p-ERK/ERK. (**C**) Protein level of p-p53 and p53 were measured by western blot analysis. (**D**) The activity of p53 was calculated by quantitation of p-p53/p53. (**E**) Relative mRNA expression levels of p53 gene were detected by quantitative real-time PCR. Each result shown represents the mean ± SEM taken from at least 3 independent experiments. * *p* < 0.05, ** *p* < 0.01, *** *p* < 0.001.

To investigate the mechanism that modulates DUSP6 expression after TDI exposure, the expression levels of upstream and downstream genes of DUSP6 were analyzed. Among the genes that modulate the expression of DUSP6, p53 regulates transcription of DUSP6 by binding the promoter region of the DUSP6 gene [27,30]. To confirm the regulatory effect of intracellular p53 by TDI exposure, various concentrations of TDI were added to BEAS-2B cells. Our results showed that the expression level and activity of p53 increased after the TDI exposure. The mRNA level of p53 increased 1.7-fold after 500 μM of TDI compared to the control (Figure 4E). TDI treatment also increased the phosphorylated form of p53 by 2.5-fold (Figure 4C,D). We also investigated the effects of TDI on ERK1/2 which is directly inactivated by DUSP6. The results showed that the activity of ERK1/2 increased up to 5-fold after 100 μM of TDI and decreased to the original level after 200 μM and 500 μM of TDI, showing opposite patterns to DUSP6 expression after TDI treatment (Figure 4A,B).

### 2.3. TDI Induces Cytotoxicity but Attenuates Apoptosis Regulated by ERK1/2

**Figure 5:**
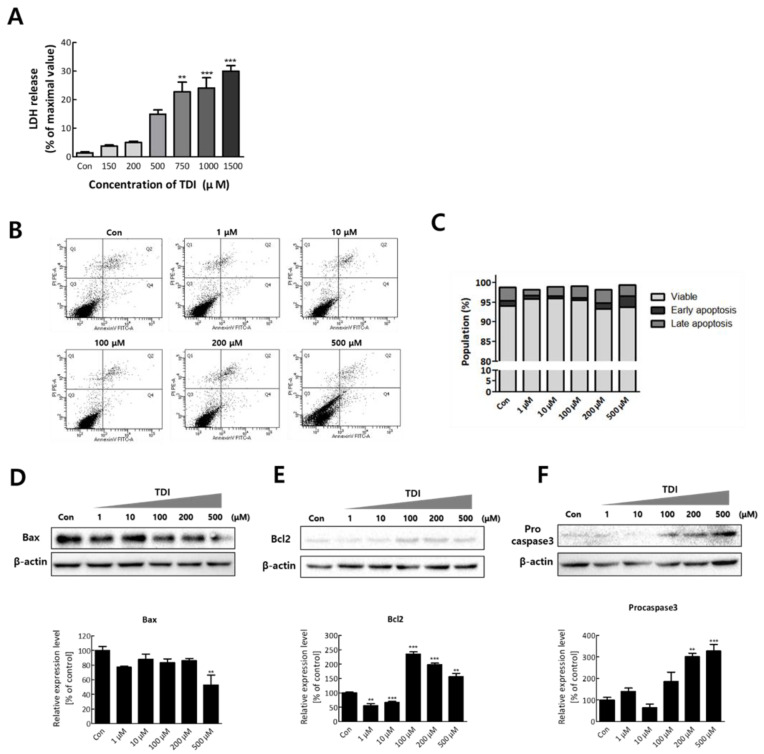
TDI induces cytotoxicity but inhibits apoptosis. (**A**) BEAS-2B cells were treated with various concentrations of TDI (150–1500 μM) for 24 h and the intensity of released LDH was detected. (**B**) Cells were incubated with TDI for 24h and harvested to analyze apoptosis rate through FACS analysis. (**C**) The percentage of cells corresponding to each stage (viable, early apoptosis, late apoptosis) was calculated and represented in a graph. The expression level of (**D**) Bax, (**E**) Bcl2, and (**F**) Procaspase3 proteins was analyzed after treatment with various concentrations of TDI (1–500 μM) for 24h. Total proteins were isolated and subjected to immunoblotting with antibodies against Bax, Bcl2, Procaspase3, or β-actin, and quantitative protein expression was calculated. Each result shown represents the mean ± SEM taken from at least three independent experiments. ** *p* < 0.01, *** *p* < 0.001.

To confirm the cytotoxic effect of TDI on BEAS-2B cells, a lactate dehydrogenase (LDH) leakage assay was conducted after TDI treatment. LDH is a cytosolic enzyme that exists in many different cell types, and it is released into the cell culture medium upon damage to the plasma membrane [31]. Therefore, the intensity of released LDH can be used as an indicator of dead cells. Dead cells after 1500 μM of TDI exposure were significantly increased (30.03 ± 1.92% of the control, *n* = 3, *p* < 0.001) (Figure 5A). In Figure 1B (24-hour), cell viability showed a tendency to increase up to 1000 μM and then decrease above 1000 μM. In Figure 1C,D (3 and 7 days), the cell viability increased under 100 μM and then decreased above it. Increase of viable cells indicates that the number of cells increased through proliferation is greater than that of dead cells. Similarly, at high concentrations of TDI, the cell viability appears to decrease because the number of dead cells is greater than the number of cells increased by proliferation. A previous study reported that low concentration of TDI triggers cell growth, while high concentration of TDI has cytotoxicity [32]. After confirming the cytotoxicity of TDI, we investigated whether cells died through apoptosis. The results showed that there were no significant differences in the apoptosis rate after TDI exposure compared to the control group (Figure 5B,C). To validate that TDI exposure did not affect apoptosis, the protein level of apoptosis-related genes was analyzed. Bax, Bcl2, and Caspase3, known as the main regulators of apoptotic response, are considered as the representative factors for apoptosis [33]. The expression level of Bax was decreased by 0.6-fold compared to the control group at 500 μM of TDI (Figure 5D). Expression levels of Bcl2 and Procaspase3 were increased at 100–500 μM of TDI (Figure 5E,F). The results showed that the expression levels of Bax and Bcl2 showed an inverse correlation consistent with the results of previous studies.

### 2.4. Down-Regulation of TDI-Induced DUSP6 Expression by TRPA1 Channel Inhibition

**Figure 6:**
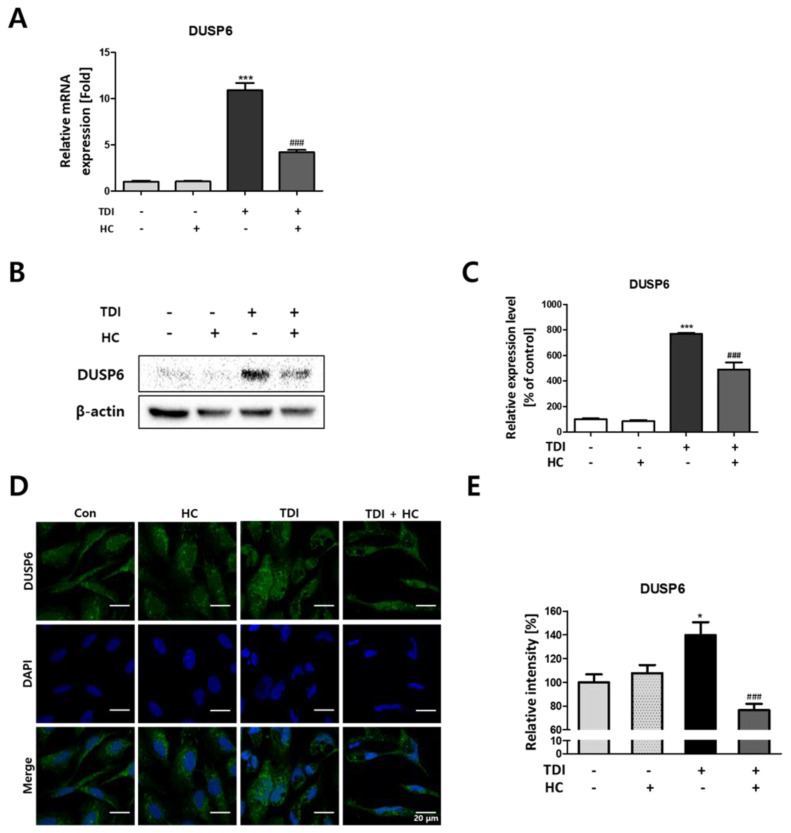
Modulatory effect of transient receptor potential ankyrin-1 (TRPA1) on TDI-induced DUSP6 expression. BEAS-2B cells were pre-treated with 20 μM HC-030031 or the vehicle and then 500 μM of TDI. (**A**) The gene expression level of DUSP6 was measured using quantitative real-time PCR (qRT–PCR). (**B**) Total proteins were isolated and immunoprecipitated with antibodies against DUSP6 and β-actin. (**C**) Quantitative protein expression of DUSP6/β-actin was calculated and represented as a graph. (**D**) Intracellular DUSP6 and the nucleus were stained by CFL 488 and DAPI, respectively. (**E**) The relative fluorescence intensity of DUSP6 was calculated and represented in a graph. Each result shown represents the mean ± SEM taken from at least three independent experiments. (* *p* < 0.05, *** *p* < 0.001) and TDI treated group (### *p* < 0.001). HC: HC-030031.

The transient receptor potential ankyrin 1 (TRPA1) channel which is Ca^2+^ permeable nonselective cation channel is known to be activated by exogenous stimuli that may produce cellular damage such as cold, heat, and mechanical stimuli [28]. Recent research showed that the TRPA1 is the primary molecular transducer through which TDI causes respiratory irritation [29]. To investigate whether TDI-induced DUSP6 is regulated through activation of TRPA1, receptor cells were treated with TDI and HC-030031, known as a TRPA1 specific antagonist. Our results showed that the increased mRNA and protein level of DUSP6 by TDI was decreased by co-treatment of HC-030031 and TDI (Figure 6). Gene expression of DUSP6 increased by 10-fold after the TDI treatment but decreased by 5-fold after co-treatment of TDI and HC-030031 (Figure 6A). The protein level also showed a similar tendency as TDI-induced DUSP6 expression was attenuated by co-treatment of TDI and the TRPA1 antagonist (Figure 6B,C). Intracellular DUSP6 intensity analyzed by ICC was also suppressed by co-treatment of TDI and the TRPA1-antagonist HC-030031 compared to TDI treated group (Figure 6D,E).

## 3. Discussion

Toluene diisocyanate (TDI) is a highly reactive carcinogenic compound used in manufacturing industries such as automobiles, furniture, and packaging materials [5]. TDI is a well-known causative agent of occupational asthma [2,4,6,34,35]. In previous studies on animals, TDI was not toxic by the oral or dermal route, but toxic and irritating by inhalation [2]. TDI is mainly exposed to the respiratory tract due to its volatilization characteristics [36,37]. Lung- and bronchial-derived BEAS-2B cells have been used as an in vitro model for the study of inhaled hazardous chemicals such as cigarette smoke and 2-hydroxyethyl methacrylate (HEMA) [38,39]. Therefore, the lung-derived BEAS-2B cell line was considered a suitable in vitro model and used for this study. While many studies which addressed the pathogenesis of TDI-induced occupational asthma have been actively conducted, the research on the molecular mechanisms of TDI exposure has not yet been properly investigated [1,16,17,35,40].

Transcriptomic analysis enables prediction of the intracellular mechanisms induced by TDI through selecting DEGs after TDI exposure and comparing the similarities and interactions between them [24]. Therefore, in this study, the total mRNA expression level was investigated after TDI exposure of human bronchial epithelial cells. For hierarchical clustering analysis, significantly changed factors were selected among the DEGs whose expression was commonly changed with both the low and high concentration of TDI (Figure 2A). Generally, the score cut-off for the confidence of interaction has been set at 0.4 or 0.5 in various studies of PPI analysis [41,42,43]. Our results showed a complicated web for protein interactions when the confidence score cut-off was 0.5 (Figure 2B). To analyze the interaction of tightly controlled factors for TDI exposure, the cut-off criterion was raised to 0.7 and re-analyzed (Figure 2C). Generally, nodes in the center of the network are classified as key factors in the PPI network. Our data showed that one of the nodes at the center of the network is DUSP6. Through the hierarchical clustering heatmap and PPI analysis, it was confirmed that DUSP6 is a major response factor that is regulated by TDI exposure (Figure 2). During verification of DUSP6 expression *in vitro*, we determined that the mRNA and protein expression level of DUSP6 increased in a dose-dependent manner, which is consistent with the previous transcriptomics’ analysis results (Figure 3A–F).

Our data showed that mRNA and protein expression of DUSP6 was up-regulated by TDI treatment in BEAS-2B cells. According to previous studies, p53, one of the upstream genes of DUSP6 is known as a transcriptional regulator of DUSP6. DUSP6 is also known to induce dephosphorylation by direct binding to ERK1/2. Previous studies have shown that TDI can induce genotoxic effects and that p53 is activated by various genotoxic stresses [2,13,44]. Therefore, we hypothesized that up-regulation of DUSP6 may be induced through up-regulation of p53 by TDI and may result in dephosphorylation of ERK1/2. Our results first showed that mRNA expression and phosphorylation of p53 increased in a dose-dependent manner (Figure 4C–E). In addition, ERK1/2, a downstream target of DUSP6, is directly inactivated by TDI treatment [25]. Our results indicated that the activation level of ERK1/2 was opposite to the expression level of DUSP6 after TDI treatment (Figure 4A,B). These results suggest two points; (1) TDI-induced up-regulation of DUSP6 may have been affected by the expression and activation of p53 (2) TDI increases intracellular DUSP6 expression but repressed the ERK1/2 activation, one of the negative downstream targets of DUSP6.

TRPA1 is an ion channel that is expressed in many mammalian cells and is known as a sensor to chemical or mechanical stress [45,46]. In recent studies, the TRPA1 receptor is activated by many irritant compounds such as formalin, hydrogen peroxide, and allyl isothiocyanate indicating that TRPA1 receptor acts as a chemo-sensor in the human body [45,47,48,49,50]. A previous study also indicated that TRPA1 acts as a major signal enhancer, inducing irritation caused by TDI exposure [29]. Therefore, in this study, we aimed to determine whether DUSP6, which acts as a key factor in the toxicity mechanism of TDI, is regulated by the TRPA1 receptor. Since HC-030031 is a well-known selective antagonist of the TRPA1 receptor, intracellular expression of DUSP6 was investigated after the co-treatment of TDI and HC-030031. Our results show that the mRNA and protein expression levels of DUSP6 induced by TDI are attenuated by HC-030031 (Figure 6A–E). These results confirmed that the TRPA1 receptor acts as a major detector for TDI exposure which is consistent with recent research that showed that TDI exposure activates TRPA1 to induce an intracellular response.

LDH released from damaged cells is used as an indicator for cytotoxicity [31]. We used LDH as a measure of cell death to determine the cytotoxic effect of TDI. The data showed that cell death was increased in a dose-dependent manner (Figure 5A). Generally, when hazardous chemicals are exposed to cells, cell death is caused by toxicological mechanisms involved in immunological damage, malignancy, necrosis, and oxidative damage [51]. In the cells that become abnormal by exposure to toxic chemicals, cell death is induced by apoptosis, oxidative stress, DNA damage, and inhibition of energy metabolism [52,53,54]. ERK1/2 is a member included in the MAPK superfamily that regulates cell survival [55]. Previous studies have shown that ERK1/2 induces the activation of some pro-apoptotic proteins and inhibits cell survival signals [56]. Therefore, to confirm whether apoptosis is down-regulated by the inhibition of ERK1/2, we investigated the relevance between them through apoptosis analysis. Our results confirmed that TDI exposure induces cytotoxicity but not through the apoptotic response (Figure 5B,C).

To confirm the effect of TDI exposure on apoptosis-related factors, the protein level of apoptosis-related genes was analyzed. Caspase3, the convergence point of the apoptotic signaling pathway, plays a key role in various apoptotic signaling pathways. Once Procaspase3 is cleaved to Caspase3, an apoptotic pathway is initiated by coordinating the destruction of cellular structures such as DNA fragmentation or degradation of cytoskeletal proteins [57]. Bax and Bcl2 have an opposite effect on apoptosis. Bax can activate some small molecules to enter into the cytoplasm, resulting in cell apoptosis [58,59]. Bcl2 binds to and inactivates Bax and other pro-apoptotic proteins, thereby inhibiting apoptosis [58,60]. Our results showed that the protein expression level of pro-apoptotic genes was decreased by TDI while that of anti-apoptotic genes was increased (Figure 5D–F). Inhibition of the apoptotic response is of great importance when understanding the mechanism of TDI toxicity. Inhibition of the apoptotic process provides cells with more opportunity for the accumulation of gene mutations that can lead to carcinogenic properties such as invasiveness and angiogenesis [61]. The apoptosis pathway is also generally inhibited in various cancer cell lines [62]. Therefore, TDI may contribute to the inhibition of apoptosis and allow cells to be more cancerous. The cells that obtained the cancerous characteristics may result in cell death through biological processes related to DNA damage, gene instability, and the absence of intercellular interactions [63]. In the result of LDH assay, cells died in a dose-dependent manner of TDI (Figure 5A), whereas overall cell viability analyzed through WST assay (Figure 1A) did not decrease and even increased after TDI treatment. The abnormal increase of cell proliferation induced by TDI can be considered as one of the carcinogenic hallmarks. Accordingly, our results suggest that the increase in abnormal cells due to inhibition of apoptosis after the TDI exposure may lead to cell death.

In this study, the toxic mechanism for TDI exposure was investigated focusing on the DUSP6 gene, a key molecule modulated by TDI treatment. Our results confirmed that the intracellular expression level of DUSP6 was increased by TDI treatment, and the activity of ERK1/2 showed a reverse correlation with the expression level of DUSP6. The expression and activation level of p53 was also significantly modulated by TDI treatment. In addition, our results showed that the TDI induces a cytotoxic effect on BEAS-2B cells but inhibits the apoptotic response, which may suggest that TDI can produce cancerous properties on BEAS-2B cells. We also confirmed that the TDI-induced modulated genes including DUSP6 are regulated by the TRPA1 receptor. Taken together, our results determined the mechanism of toxicity for respiratory exposure of TDI. Our results can provide important insights into the human effects of TDI and can offer prospects for research on reducing the toxic effects of TDI exposure.

## 4. Materials and Methods

### 4.1. Chemicals and Reagents

The Toluene 2,4-diisocyanate was purchased from Junsei Chemical Co., dimethyl sulfoxide (DMSO), HC-030031, protease inhibitor cocktail, phosphatase inhibitor cocktail 2/3, and sodium dodecyl sulfate (SDS) were purchased from Sigma-Aldrich Chemical (St. Louis, MO, USA). Antibody against anti-DUSP6 (ab238512) was purchased from Abcam (Cambridge, MA, USA). Antibodies against anti-p-ERK1/2 (9101S), and anti-p-p53 (9284S) were purchased from Cell Signaling Technology (Beverly, MA, USA). Antibodies against anti-p53 (sc-126, anti-ERK1/2 (sc-514302), anti-Caspase3 (sc-7148), anti-Bax (sc-526), anti-Bcl2 (sc-7382), β-actin (sc-47778), HRP-conjugated anti-rabbit IgG (sc-2357), HRP-conjugated anti-mouse IgG (sc-516102), and mouse anti-rabbit IgG-CFL 488 (sc-516248) were purchased from Santa Cruz biotechnology (Santa Cruz, CA, USA). RNeasy Mini Kit was from Qiagen (Qiagen, Hilden, Germany), and the lactate dehydrogenase (LDH) assay kit was from DYNEBIO (Seoul, Korea).

### 4.2. Cell Culture and Treatments

Human bronchial epithelial cells (BEAS-2B) were purchased from the American Type Culture Collection (ATCC, Manassas, VA, USA). The cells were cultured in the Bronchial epithelial basal medium (BEBM, Lonza, Switzerland) with cell growth supplements (BEGM, Lonza, Switzerland) in a humidified incubator with 5% CO_2_ at 37 °C. The medium was replaced with fresh media every three days. For further experiments, BEAS-2B cells were seeded at a density of 2 × 10^4^ cells/cm^2^ and used for experiments when the confluency reached 70–80%. For treatment of TDI, cell medium was changed to Roswell Park Memorial Institute Medium (RPMI 1640, welgene, Daegu, Korea) containing 1% of fetal bovine serum (FBS, welgene, Daegu, Korea), 1% of sodium pyruvate, and 1% of penicillin/streptomycin (welgene, Daegu, Korea). To administer the TDI and HC-030031 to cells, TDI and HC-030031 were diluted in DMSO and added for 24 h.

### 4.3. Cell Viability Assay

Cell viability was measured using an WST assay. Cells were seeded into 96-well plates at 1 × 10^4^ cells/well in 200 μL of BEBM media. After the cell stabilization for 24 h, various concentrations of TDI (0.1–5000 μM) were added for 4 h, 24 h, 3 days, and 7 days. Cell culture medium with TDI was replaced with serum-free RPMI 1640 medium containing the EZ-CYTOX reagent (DOGEN, Seoul, Korea) and the cells were incubated for 2 h to allow the substance to react. The absorbance of each well was measured at 450 nm using a microplate reader (Molecular Devices, San Jose, CA, USA). The cell viability of the toluene diisocyanate-treated group was quantified compared to the vehicle-treated control group.

### 4.4. mRNA Extraction

For extraction of total RNA, BEAS-2B cells were seeded into 6-well plates at a density of 4 × 10^4^ cells/well. Total RNA was prepared using an RNA extraction kit (Qiagen, Hilden, Germany) according to the manufacturer’s instructions. Isolated total RNA was dissolved in RNase-free water and quantified by Nanodrop-2000 (Thermo Fisher Scientific, Waltham, MA, USA). The purity of total RNA was examined by a standard of the optical density ratio at 260 nm and 280 nm which is ranged between 1.8 and 2.0. The integrity of the total extracted RNA was confirmed by agarose gel electrophoresis at 50 V for 30 min.

### 4.5. 3’ mRNA Quantification Sequencing (3’ mRNA Quan-Seq)

The mRNA-quantification sequencing was performed by E-Biogen Inc. (Seoul, Korea). 10 μM of total RNA was extracted and prepared for library creation. For each RNA sample, the library was constructed using a QuantSeq 3’ mRNA-Seq Library Prep Kit FWD for Illumina (Lexogen, Vienna, Austria) following the manufacturer’s instructions. Quantitation and visualization of data were conducted by Excel-based Differentially Expressed Gene Analysis (ExDEGA, E-Biogen, Inc., Seoul, Korea).

### 4.6. Quantification Real-Time Polymerase Chain Reaction (qRT-PCR)

Total RNA with a mass of 2 μg was converted to complementary DNA (cDNA) by Reverse Transcriptase (ELPIS-BIOTECH, Daejeon, Korea). To quantify gene expression, cDNA was amplified with the SYBR Green PCR Master Mix (KAPA, MA, USA) and a pair of primers through qRT-PCR (CFX Connect™ Real-Time PCR Detection System; Bio-Rad, Hercules, CA, USA). The amplification was performed using the following consisted conditions: initial denaturation at 95 °C for 3 min, followed by 40 cycles of denaturation at 95 °C for 10 s, annealing at 60 °C for 10 s, and extension at 72 °C for 10 s. After the amplification reaction, melting curve analysis was immediately conducted for each replicated well to assess the specificity of the PCR reaction.

### 4.7. Western Blot Analysis

Total proteins were extracted from BEAS-2B cells using RIPA buffer (Bio Solution, Seoul, Korea) with phosphatase-inhibitor cocktail 2/3 (Sigma-Aldrich Chemical, St. Louis, MO, USA) and protease inhibitor (Sigma-Aldrich Chemical, St. Louis, MO, USA) on ice. The concentration of protein was determined by a BCA protein assay kit (Thermo Scientific, Waltham, MA, USA) with albumin as a standard. Protein samples of equal concentration were separated via sodium dodecyl sulfate polyacrylamide gel electrophoresis (SDS-PAGE) and the protein was transferred to polyvinylidene difluoride membranes (BioRad, Hercules, CA, USA). Membranes were blocked by 5% skim milk in TBST at room temperature for 1 h. Without washing, primary antibodies were administrated to the membrane at 4 °C overnight. After being washed five times with TBST, secondary antibodies conjugated with HRP were administered to the membrane at room temperature for 45 min. After the washing four times with TBST buffer, proteins were detected with chemiluminescence (ECL) Plus Western Blotting reagents (Amersham Bioscience, Buckinghamshire, UK) and quantified using a Bio-Rad ChemiDoc XRS system (Bio-Rad, Hercules, CA, USA). β-actin was used as an internal control to normalize the data.

### 4.8. LDH Cytotoxicity Assay

LDH release from the cell was measured using the LDH Cytotoxicity Assay kit (DYNEBIO, Seongnam, Korea). BEAS-2B cells were seeded into 96-well plates and various concentrations of TDI (150–1500 μM) were added for 24 h. Each sample was incubated with a reaction mixture containing lactate, NAD^+^, and water-soluble tetrazolium salt (WST) for 30 min at room temperature under dark conditions. Subsequently, 10 μL of supplied Stop Solution was added and mixed gently. The optical density of each well was measured at 490 nm using a microplate reader (Molecular Devices, San Jose, CA, USA). The cytotoxic value is calculated by the formula below, according to the manufacturer’s protocol.

$$\mathrm{Cytotoxicity}(\%)=\frac{\left(A-B\right)}{\left(C-B\right)}\times 100$$

A: Experiment value–background control; B: Low control–background control; C: High control–background control; Low control is untreated cells and indicates all living cells. High control, called the positive control, was treated with 10 μL of supplied Cell Lysis Solution for 5 min in the dark.

### 4.9. Fluorescein Isothiocyanate (FITC)-Annexin V and Propidium Iodide Double Staining for Apoptosis Detection

Apoptosis was detected by FITC Annexin V Apoptosis Detection Kit I (BD Biosciences, Franklin Lake, NJ, USA). Cells were harvested and washed with cold PBS buffer and resuspended in 150 μL of binding buffer. Then, 100 μL of the cell suspension (1 × 10^5^ cells) was transferred to a polystyrene tube (Falcon, BD Biosciences, San Jose, CA, USA) and stained with 10 μL of annexin V dye and 5 μL of PI. After being vortexed gently, cells were incubated for 15 min at room temperature in dark conditions. Then, 400 μL of binding buffer was added to be analyzed using FACSCalibur (BD Biosciences, Franklin Lake, NJ, USA).

### 4.10. Immunocytochemistry (ICC) Staining

Cells were seeded into 6-well plates pre-coated with poly-L-lysine (Sigma-Aldrich Chemical, St. Louis, MO, USA) and stabilized for 24 h. After fixation with 4% of formaldehyde (Sigma-Aldrich Chemical, St. Louis, MO, USA) for 10 min, cells were treated with 0.25% TritonX-100 (Sigma-Aldrich Chemical, St. Louis, MO, USA) to permeabilize all lipid bilayers for 10 min. After being washed with PBS buffer two times, cells were blocked with 1% of BSA solution at room temperature for 30 min. Primary antibody against DUSP6 was added to cells at 4 °C overnight. Then, cells were incubated with mouse anti-rabbit IgG-CFL 488 for 1 h. Nuclei of cells were stained using DAPI. Fluorescent images were measured by confocal microscope (Carl Zeiss, Oberkochen, Germany) and quantified by ImageJ version 1.53n (National Institutes of Health, Bethesda, MD, USA).

### 4.11. Statistical Analysis

All represented values were analyzed by GraphPad Prism version 5.0 (GraphPad Software, Inc., San Diego, CA, USA) and presented as the mean ± SEM. Statistical significance was determined by one-way ANOVA with Tukey’s multiple comparison analysis and *p*-value < 0.05 was considered significant.

## 5. Conclusions

This study shows the toxic mechanism for respiratory exposure of TDI. We identified and discovered DUSP6 as a key factor for TDI exposure by using transcriptomic analysis. We verified that mRNA and protein expression of DUSP6 was up-regulated by TDI treatment. Our data showed that TDI regulates DUSP6 by up-regulation of p53 and thereby down-regulating ERK1/2 through TRPA1 receptor activation. TDI exposure also induced cytotoxicity but attenuated the apoptotic response, which suggest that it may promote the survival of cancerous cells. Our study suggests that TDI induces DUSP6 and p53 but attenuates ERK1/2 activity through TRPA1 receptor activation, thereby leading to cytotoxicity.

## Data Availability

Not applicable.
